# Risk Factors and Prevalence of *Helicobacter pylori* in Five Largest Islands of Indonesia: A Preliminary Study

**DOI:** 10.1371/journal.pone.0140186

**Published:** 2015-11-23

**Authors:** Ari Fahrial Syam, Muhammad Miftahussurur, Dadang Makmun, Iswan Abbas Nusi, Lukman Hakim Zain, Zulkhairi, Fardah Akil, Willi Brodus Uswan, David Simanjuntak, Tomohisa Uchida, Pangestu Adi, Amanda Pitarini Utari, Yudith Annisa Ayu Rezkitha, Phawinee Subsomwong, Nasronudin, Rumiko Suzuki, Yoshio Yamaoka

**Affiliations:** 1 Division of Gastroenterology, Department of Internal Medicine, Faculty of Medicine, University of Indonesia, Jakarta, Indonesia; 2 Department of Environmental and Preventive Medicine, Oita University Faculty of Medicine, Yufu, Japan; 3 Gastroentero-Hepatology Division, Department of Internal Medicine, Airlangga University Faculty of Medicine, Surabaya, Indonesia; 4 Institute of Tropical Disease, Airlangga University, Surabaya, Indonesia; 5 Division of Gastroentero-Hepatology, Department of Internal Medicine, Faculty of Medicine, University of Sumatera Utara, Medan, Indonesia; 6 Center of Gastroentero-Hepatology, Department of Internal Medicine, Faculty of Medicine, Hasanuddin University, Makassar, Indonesia; 7 Department of Internal Medicine, Santo Antonius Hospital, Pontianak, Indonesia; 8 Department of Internal Medicine, Yowari Hospital, Jayapura, Indonesia; 9 Department of Molecular Pathology, Oita University Faculty of Medicine, Yufu, Japan; 10 Department of Gastroenterology and Hepatology, Baylor College of Medicine and Michael DeBakey Veterans Affairs Medical Center, Houston, Texas, United States of America; University of Hyderabad, INDIA

## Abstract

The prevalence of *Helicobacter pylori* infection in Indonesia is still controversial and mainly investigated in the largest ethnic group, Javanese. We examined the prevalence of *H*. *pylori* infection using four different tests including culture, histology confirmed by immunohistochemistry and rapid urease test. We also analyzed risk factors associated with *H*. *pylori* infection in five largest islands in Indonesia. From January 2014–February 2015 we consecutively recruited a total of 267 patients with dyspeptic symptoms in Java, Papua, Sulawesi, Borneo and Sumatera Island. Overall, the prevalence of *H*. *pylori* infection was 22.1% (59/267). Papuan, Batak and Buginese ethnics had higher risk for *H*. *pylori* infection than Javanese, Dayak and Chinese ethnics (OR = 30.57, 6.31, 4.95; OR = 28.39, 5.81, 4.61 and OR = 23.23, 4.76, 3.77, respectively, P <0.05). The sensitivity and specificity for RUT and culture were 90.2%, 92.9% and 80.5%, 98.2%, respectively. The patients aged 50–59 years group had significantly higher *H*. *pylori* infection than 30–39 years group (OR 2.98, P = 0.05). Protestant had significantly higher *H*. *pylori* infection rate than that among Catholic (OR 4.42, P = 0.008). It was also significantly lower among peoples who used tap water as source of drinking water than from Wells/river (OR 9.67, P = 0.03). However only ethnics as become independent risk factors for *H*. *pylori* infection. Although we confirmed low prevalence of *H*. *pylori* in Javanese; predominant ethnic in Indonesia, several ethnic groups had higher risk of *H*. *pylori* infection. The age, religion and water source may implicate as a risk factor for *H*. *pylori* infection in Indonesia.

## Introduction

*Helicobacter pylori* infection has been recognized as one of the most common chronic bacterial infections in humans and associated with peptic ulcer disease, gastric adenocarcinoma, and primary gastric B-cell lymphoma [1]. The overall prevalence varies globally from one geographical region to another with occurs mainly in developing countries. Indonesia is a developing country located between South China Sea (Pacific Ocean, in North) and the Indian Ocean (in South); it is an archipelago of more than 13,600 islands with Sumatra, Papua, Kalimantan (Borneo), Sulawesi and Java as five main islands. There are around 300 distinct native ethnic groups in Indonesia, and 742 different languages which most of them belonging to the geographically dispersed Austronesian-speaking family [2]. Javanese is the largest ethnic group who comprise 40.2% of the total population, followed by Sundanese, Batak, Madurese and Betawi (Statistics Indonesia, http://www.bps.go.id/).

Table 1 is the summarizes of previous studies that examined the prevalence of *H*. *pylori* in Indonesia (Table 1). Although many researchers have investigated the prevalence in Indonesia, the results are controversial and contradictory (0–68%) [3,4] probably due to the different study populations and different tests for *H*. *pylori* diagnosis [5]. Moreover these studies mainly investigated only the largest ethnic group, Javanese [3,4,6–10]. In our previous study, we confirmed that the prevalence of *H*. *pylori* infection in Surabaya (Java island) was low, only 11.5% using five different methods to diagnose *H*. *pylori* infection [5], the data were concordance with the low age-standardized incidence rate of gastric cancer in Indonesia among Asian countries (2.8/100,000; GLOBOCAN2012, http://globocan.iarc.fr/). We also found that the highest prevalence of *H*. *pylori* was observed in patients from the Chinese Indonesian population instead of patients from the Javanese population. Another our study also found unexpected result about the prevalence of *H*. *pylori* infection in minor group in North Sulawesi. The overall *H*. *pylori* prevalence was only 14.3% for adults and 3.8% for children [11]. However our results cannot be generalized across Indonesia due to the difference of host factor and environmental condition. Further investigation from all Indonesia is necessary to elucidate the reasons of low gastric cancer rate in Indonesia.

**Table 1:** Summary previous *Helicobacter pylori* prevalence studies in Indonesia.

Author	Study period	Area	n	Average age (range)	Test	Positive rate
Syam AF [6]	2001	Jakarta	63	42.4 (16–73)	Stool antigen	66.7% (42/63)
					RUT	4.8% (3/63)
					Histology	11.1% (7/63)
Tokudome S [10]	2003	Yogyakarta	91	48.0 for men	UBT	4% in men and 0% in women
				46.6 for women	Serum antibody	5% in men and 4% in women
Tokudome S [3]	2005	Semarang	171	57.4 for men	UBT	0% in men and 0% in women
				49.2 for women	Serum antibody	2% in men and 2% in women
Syam AF [14]	2003–2004	6 cities	550	44.98 (15–82)	Histology	10.2% (56/550)
Saragih JB [7]	1998–2005	Jakarta	2903	no information	Histology	12.8% (52/407) in 1998
						2.9% (50/403) in 2005
Aulia D [8]	2007	Jakarta	70	47.6 (18–79)	Histology	5.7% (4/70)
Abdullah M* [4]	1998–1999	Jakarta	125	50.3 (18–82)	RUT	68% (85/125) in the antrum4% (5/125) in the corpus
					Culture	
					Histology	
Arinton IG [9]	2005	Purwokerto	81	56.8 (45–75)	PCR	41.9% (34/81)
Zhao Y [15]	2007	Mataram	294	34.0 (6–74)	UBT	11.2% (33/294)
Miftahussurur M [11]	2011–2012	Manado	251 Adults	46.2 (14–88)	Urine test	Adults 14.3% (36/251)
			131 Children	8.47 (6–12)		Children 3.8% (5/131)
Miftahussurur M [5]	2012	Surabaya	78	49.1 (14–77)	Urine test	5.1% (4/78)
					RUT	9.0% (7/78)
					Culture	6.4% (5/78)
					Histology + IHC	7.7% (6/78)
					Overall	11.5% (9/78)

UBT, urea breath test; PCR, polymerase chain reaction; RUT, rapid urease test

*This study tested for *H*. *pylori* by histology, culture, and rapid urease test

The presence of *H. pylori* in saliva, dental plaque [12], and feces [13] indicated that person-to-person spreading is probably a major transmission mechanism of *H*. *pylori*. A number of studies have found poor hygiene standards, crowded households and deficient sanitation are important to both acquisition of infection in childhood and spreading of this disease. Lower social economic status, non-filtered water, and smoking to be a risk factor for *H*. *pylori* [16]. On the other hand, the improvement of hygiene conditions has significantly decreased the prevalence of this infection in many parts of North America and Europe [17]. In Japan, the prevalence of *H*. *pylori* infection was higher among individuals born before 1950 and lower in those born thereafter; the data indicated a rapid change in the sanitary conditions and standard of living in Japan after the World War II, and clean public water systems were introduced in Japan in the 1950s. Therefore, sanitary conditions, such as a full equipment rate of water and sewage, are considered to be important factors for *H*. *pylori* infection [18].

To our knowledge, very few reports had investigated *H*. *pylori* in non-Javanese ethnics [11,14,15] and no report had examined the prevalence of *H*. *pylori* infection from several islands in Indonesia using same methods. In this study, we examined the prevalence of *H*. *pylori* infection in five largest islands using four different tests. We also identified and analyzed environmental factors on different ethnics in Indonesia.

## Materials and Methods

### Study population

**Fig 1:**
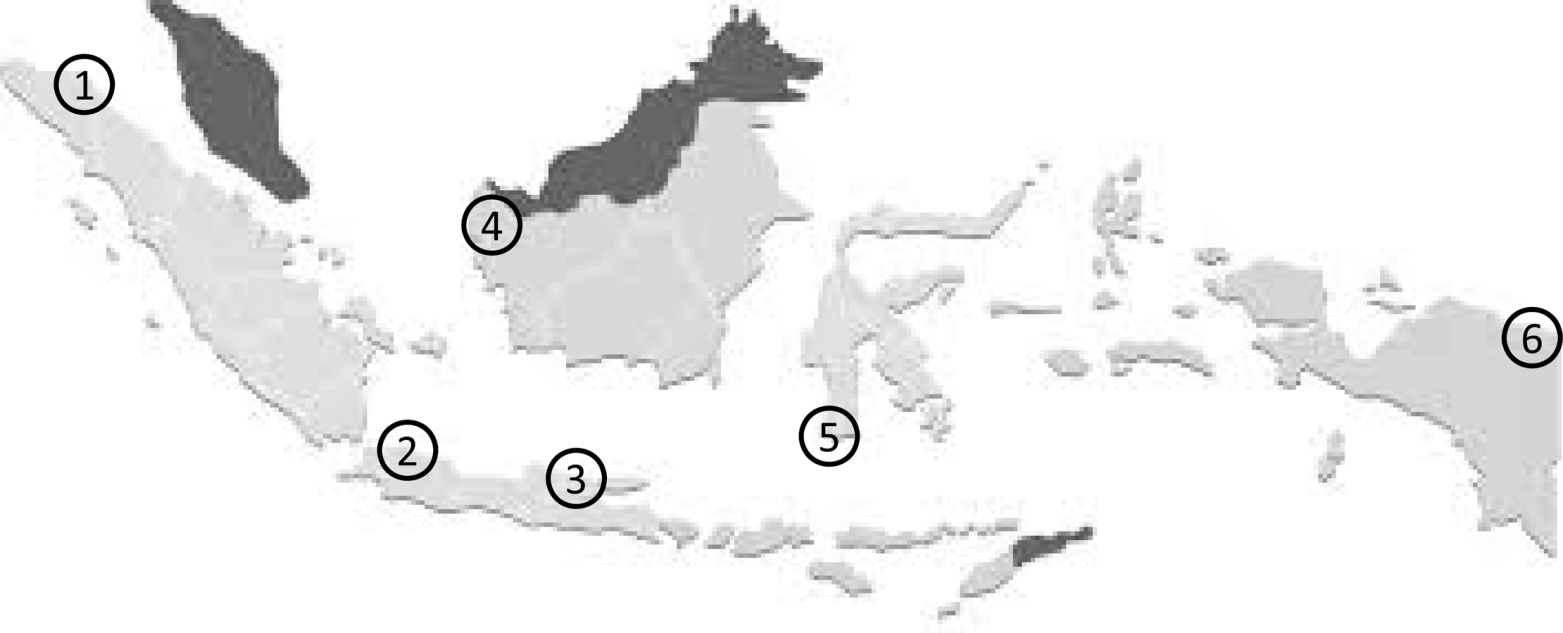
Map of collecting area in Indonesia. A total of 267 consecutive patients were obtained biopsy specimen at the five largest islands in Indonesia; (1) Medan (Sumatera island), (2) Jakarta (Java island), (3) Surabaya (Java island), (4) Pontianak (Borneo island), (5) Makassar (Sulawesi island), and Jayapura (Papua island).

We performed prospective study from January 2014 until February 2015. The survey took place on Jakarta (n = 31) and Surabaya (n = 50) in Java island, Jayapura (n = 21) in Papua island, Makassar (n = 30) in Sulawesi island, Pontianak (n = 64) in Borneo island and Medan (n = 71) in Sumatera island (Fig 1). Experienced endoscopists collected four gastric biopsy specimens during each endoscopy session: three samples from the lesser curvature of the antrum approximately 3 cm from the pyloric ring and one sample from the greater curvature of the corpus. To minimize the potential bias, we used the same experienced pathologist (TU) performed the experiments, who also performed experiments for Myanmar, Vietnam, Bhutan, Dominican Republic and Indonesia [5,19–23]. Biopsy specimens for culture were immediately placed in transport media at -20°C, and stored at -80°C within a day of collection until used for culture testing. Three antral specimens were used for *H*. *pylori* culture, rapid urease test (RUT), and histological examination. One corporal specimen was used for histological examination. Peptic ulcers and erosive gastritis were identified by endoscopy. Written informed consent was obtained from all participants, and the study protocol was approved by the Ethics Committee of Dr. Cipto Mangunkusumo Teaching Hospital (Jakarta, Indonesia), Dr. Soetomo Teaching Hospital (Surabaya, Indonesia), Dr. Wahidin Sudirohusodo Teaching Hospital (Makassar, Indonesia) and Oita University Faculty of Medicine (Yufu, Japan).

### *H*. *pylori* infection status

To maximize diagnostic accuracy, *H*. *pylori* infections were diagnosed based on the combined results of three methods from four different tests; culture, histology confirmed by immunohistochemistry (IHC) and rapid urease test (CLO test, Kimberly-Clark, USA). For *H*. *pylori* culture, one antral biopsy specimen was homogenized and directly inoculated onto Mueller Hinton II Agar medium (Becton Dickinson, NJ, USA) supplemented with 7% horse blood without antibiotics. The plates were incubated for up to 10 days at 37°C under microaerophilic conditions (10% O_2_, 5% CO_2_, and 85% N_2_). *H*. *pylori* were identified on the basis of colony morphology, Gram staining results, and positive reactions for oxidase, catalase, and urease. Isolated strains were stored at -80°C in Brucella Broth (Difco, NJ, USA) containing 10% dimethylsulfoxide and 10% horse serum.

All biopsy materials for histological testing were fixed in 10% buffered formalin and embedded in paraffin. Serial sections were stained with hematoxylin and eosin as well as May–Giemsa stain. Samples with bacterial loads greater than or equal to grade 1 by updated Sydney system were considered positive for *H*. *pylori*. IHC was also performed as previously described [24]. Briefly, after antigen retrieval and inactivation of endogenous peroxidase activity, tissue sections were incubated with α-*H*. *pylori* antibody (DAKO, Denmark) overnight at 4°C. After washing, the sections were incubated with biotinylated goat antirabbit IgG (Nichirei Co., Japan), followed by incubation with an avidin-conjugated horseradish peroxidase solution (Vectastain Elite ABC kit; Vector Laboratories Inc., Burlingame, CA, USA). Peroxidase activity was detected using an H_2_O_2_/diaminobenzidine substrate solution.

### Statistical analysis

Discrete variables were tested using the chi-square test; continuous variables were tested using the Mann-Whitney *U* and *t*-tests. A multivariate logistic regression model was used to calculate the odds ratios (OR) of the clinical outcomes that included age, sex, *H*. *pylori* infection status, demographic and environment information. All determinants with P values of < 0.10 were entered together into the full logistic regression model, and the model was reduced by excluding variables with P values of > 0.10. The OR and 95% confidence interval (CI) were used to estimate the risk. A P value of < 0.05 was accepted as statistically significant. The SPSS statistical software package version 18.0 (SPSS, Inc., Chicago, IL) was used for all statistical analyses.

## Results

### Prevalence of *H*. *pylori* infection and accuracy several tests

A total of 267 patients with dyspeptic symptoms (143 female and 124 male; mean age of 47.5 ± 14.6 years; range, 17–80 years) were recruited including 39 patients aged ≤29 years, 40 patients aged 30–39 years, 67 patients aged 40–49 years, 57 patients aged 50–59 years, and 64 patients aged ≥60 years. Based on ethnic group they were consisted of 70 Batak subjects, 54 Chinese Indonesian, 42 Javanese, 30 Buginese, 40 Dayak, 21 Papuan, three Madurese, two Acehnese, two Sundanese, one Banjarese, one Balinese, and one Ambonese subject. Among three tests, RUT showed higher positive rate compared with other tests (both P <0.001).

**Fig 2:**
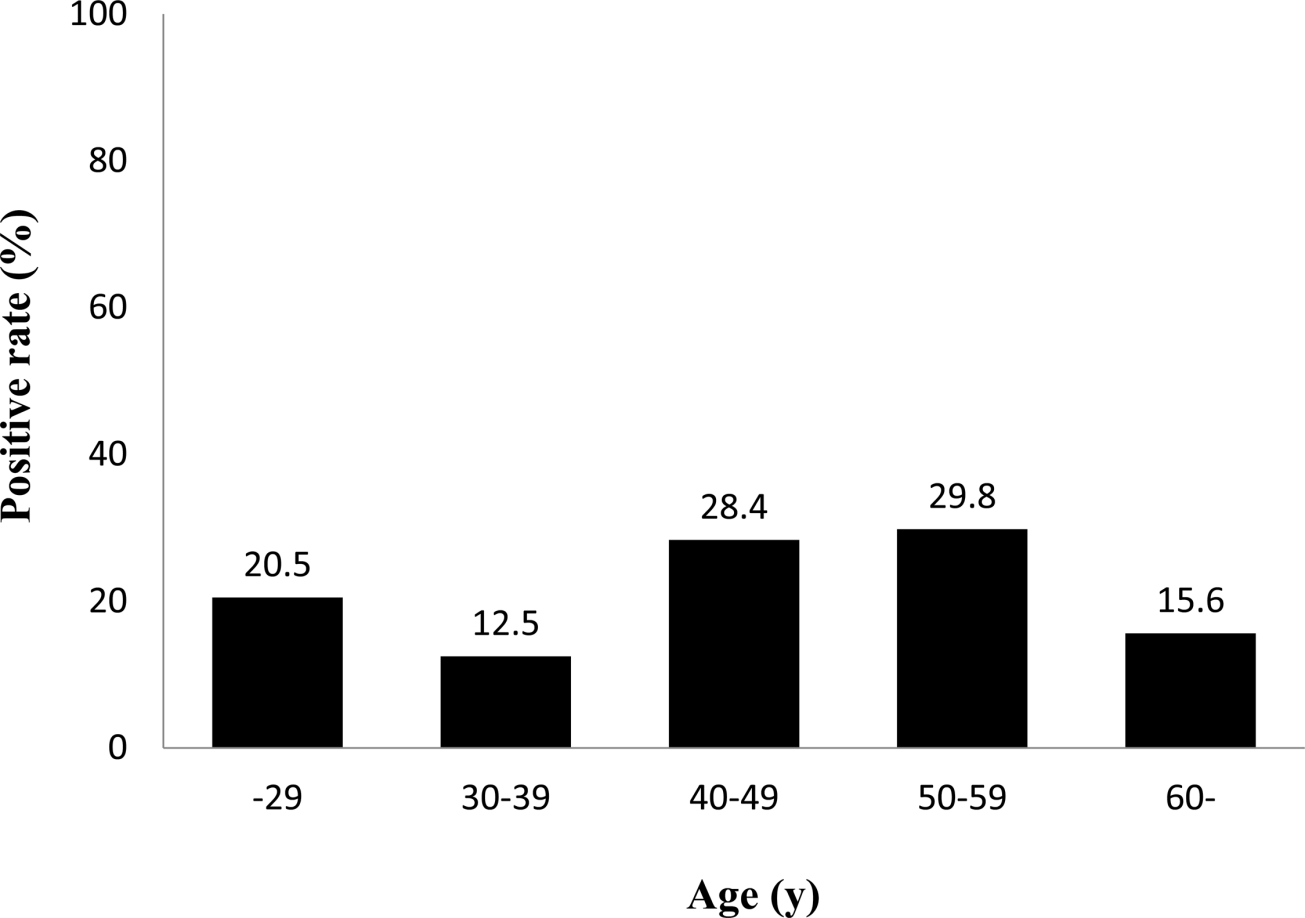
Prevalence of *Helicobacter pylori* infection in Indonesia by age group. Three different methods were used to test for *H*. *pylori* infection, including culture, histology confirmed by immunohistochemistry and rapid urease test. Patients were considered negative for *H*. *pylori* when all test results were negative; *H*. *pylori*-positive status required at least one positive test result.

Table 2 shows *H*. *pylori*-positive rates for each test. Thirty-two patients were positive by all three tests. Fourteen patients were positive only by the RUT. Three and two patients were positive only by histology and culture, respectively. Using histology confirmed IHC as a gold standard, the sensitivity and specificity of RUT and culture were 90.2%, 92.9% and 80.5%, 98.2%, respectively. Negative predictive value (NPV) and positive predictive value (PPV) were 98.1%, 69.8% and 96.5%, 89.2%, respectively. Overall accuracy rates were 92.5% and, 95.5%, respectively. Using histology confirmed IHC, the prevalence of *H*. *pylori* infection was 15.4% (41/267), whereas using culture it was 13.9% (37/267) (S1 Table). However when patients were considered to be *H*. *pylori* positive in case at least one test showed positive, the prevalence of *H*. *pylori* infection was 22.1% (59/267). In the subsequent analyses, patients were considered to be negative for *H*. *pylori* infection when all test results were negative, whereas patients with at least one positive test result were considered positive for *H*. *pylori* infection (Fig 2).

**Table 2:** Prevalence of *Helicobacter pylori* infection in each diagnostic test n (%).

	-29	30–39	40–49	50–59	60-	Total
n	39	40	67	57	64	267
RUT	7 (17.9)	5 (12.5)	18 (26.9)	13 (22.8)	10 (15.6)	53 (19.9)
Culture	7 (17.9)	4 (10.0)	10 (14.9)	8 (14.0)	8 (12.5)	37 (13.9)
Histology confirmed by IHC	6 (15.4)	4 (10.0)	13 (19.4)	12 (21.1)	6 (9.4)	41 (15.4)
Positive if at least one test result positive	8 (20.5)	5 (12.5)	19 (28.4)	17 (29.8)	10 (15.6)	59 (22.1)

### Symptoms, endoscopic findings and *H*. *pylori* infection rate

Epigastric pain and bloating were the highest symptoms. There were no significant difference between positivity of *H*. *pylori* infection with variables relating to gastrointestinal symptoms and past illness history (P = 0.36 and P = 0.74, respectively) (Table 3). In endoscopic diagnosis, gastric and duodenal ulcer was found among 4 cases (1.5%) and 29 cases (10.9%), respectively. Seven patients have both gastric and duodenal ulcer (2.6%). Gastric cancer was found in 1 case (0.4%); however *H*. *pylori* infection was negative. Among 19 subjects with normal endoscopy, 5 (26.3%) were infected with *H*. *pylori*. There was no difference the proportion of patients were infected with *H*. *pylori* in gastritis (44, 18.2%) and peptic ulcer group (10, 25.0%) (P = 0.66).

**Table 3:** Gastrointestinal symptoms and past illness history classified by *H*. *pylori* results.

	n	*H*. *pylori-*negative (n = 208)	*H*. *pylori*-positive (n = 59)
Symptoms			
• Epigastric pain	156	120 (57.7%)	36 (61.0%)
• Heart burn	10	8 (3.8%)	2 (3.4%)
• Abdominal pain	22	21 (10.1%)	1 (1.7%)
• Bloating	37	26 (12.5%)	11 (18.6%)
• History of hematemesis/melena	16	11 (5.3%)	5 (8.5%)
• Nausea/vomiting	18	14 (6.7%)	4 (6.8%)
Illness history			
• Diabetes mellitus	21	17 (8.2%)	4 (6.8%)
• Hypertension	50	39 (18.8%)	11 (18.6%)
• dyslipidemia	4	4 (1.9%)	0 (0.0%)
• Asthma	2	1 (0.5%)	1 (1.7%)
• Hepatitis/Chronic liver disease	6	1 (0.5%)	5 (8.5%)
• Tuberculosis	2	2 (1.0%)	0 (0.0%)

### Ethnic groups and *H*. *pylori* infection rate according to demographic, sanitation and socio-cultural factors

There were significant difference on the prevalence of *H*. *pylori* infection related with ethnics group (P <0.001). The highest prevalence of *H*. *pylori* infection was Papuan patients; nine of 21 (42.9%) were positive for *H*. *pylori*. Among 70 Batak patients, 28 (40.0%) was positive for *H*. *pylori*. *H*. *pylori* infection was found in 11 of 30 Buginese (36.7%) patients. On the other hand, seven of 54 (13.0%) Chinese were positive for *H*. *pylori* infection; Chinese-Surabaya (15.4%, 4/26) was no significance difference of *H*. *pylori* infection compared to Chinese Pontianak (12.5% (3/24), P = 0.93). Three Dayak (7.5%) were positive for *H*. *pylori* infection. Among 42 Javanese patients, only one (2.4%) was positive for *H*. *pylori* infection. Madurese, Acehnese, Sundanese, Banjarese, Balinese and Ambonese patients were negative for *H*. *pylori* infection.

Table 4 shows the prevalence of *H*. *pylori* infection in the sixth largest ethnics number group according to various range age groups. Interestingly when we just analyzed Papuans, Batak and Buginese, the youngest group aged ≤29 had high prevalence of *H*. *pylori* infection contrary with the low *H*. *pylori* prevalence group (Javanese, Dayak, and Chinese). The ethnic groups also had significant difference with religion, monthly income, source of water, type of latrine, history of drugs, smoking habit and alcohol consumption (Table 5). Papuan ethnic significantly had high prevalence of Protestant, high subjects with low socio-economic (monthly family income IDR 2.500.000 = 193.31 USD), high smokers and alcohol users, but low mineral water source. On the other hand, Chinese ethnic had high prevalence of Protestant and mineral water source, but low prevalence of low socioeconomic, smokers and alcohol users.

**Table 4:** Prevalence of *Helicobacter pylori* infection in the sixth largest ethnics number group (%).

	Papuan	Batak	Buginese	Chinese	Dayak	Javanese
n	21	70	30	54	40	42
-29	2 (50.0)	4 (66.7)	2 (66.7)	0 (0.0)	0 (0.0)	0 (0.0)
30–39	2 (33.3)	1 (12.5)	1 (16.7)	1 (14.3)	0 (0.0)	0 (0.0)
40–49	2 (28.6)	11 (52.4)	2 (28.6)	1 (7.7)	2 (20.0)	1 (35.7)
50–59	2 (100)	10 (55.6)	2 (50.0)	2 (20.0)	1 (10.0)	0 (0.0)
60-	1 (50)	2 (11.8)	4 (40.0)	3 (18.8)	0 (0.0)	0 (0.0)
Total	9 (42.9)	28 (40.0)	11 (36.7)	7 (13.0)	3 (7.5)	1 (2.4%)

**Table 5:** Details of subjects classified by ethnic group (%).

Variable	Papuan	Batak	Buginese	Chinese	Dayak	Javanese
Number	21	70	30	54	40	42
Age (years)**	41 (23–63)	49.5 (24–80)	48 (22–76)	49.0 (17–77)	43.0 (18–77)	48.5 (18–70)
Body Mass Index (kg/m2)***	23.9 ± 3.26	22.0 ± 2.52	23.0 ± 3.90	23.0 ± 3.75	21.1 ± 3.64	23.3 ± 4.11
Sex (% males)	52.4	41.4	53.3	44.4	57.5	45.2
Majority religion (%)*	Protestant(100)	Protestant(75.7)	Muslim(86.7)	Protestant(35.2)	Catholic(52.5)	Muslim(90.5)
Monthly income <192.31 USD (%)*	66.6	77.1	26.7	25.9	67.5	50.0
Mineral water (%)*	57.1	38.6	70.0	75.9	22.5	30
Latrine non-toilet (%)	4.8	0.0	3.3	0.0	2.5	0.0
Smokers (%)*	28.6	31.4	13.3	3.7	45.0	19.1
Alcohol user (%)*	23.8	18.6	6.7	9.3	42.5	9.5
*H*. *pylori* positive (%)	42.9	40.0	36.7	13.0	7.5	2.4

*P <0.05

** Median (minimum-maximum)

*** Mean ± standard deviation

Adjusted OR were calculated for *H*. *pylori* infection rate with multivariate analysis (Table 6). We entered all determinants with P values of < 0.10 by bivariate analysis (age, sex, religion, ethnics and source of drinking water) into the full logistic regression model. Papuan, Batak and Buginese had higher risk for *H*. *pylori* infection than Javanese (P <0.05). Moreover the next analysis on the sixth largest ethnics in this study; Papuan, Batak and Buginese ethnics had higher risk for *H*. *pylori* infection than Javanese, Dayak and Chinese ethnics (OR = 30.57, 6.31, 4.95; OR = 28.39, 5.81, 4.61 and OR = 23.23, 4.76, 3.77, respectively, P <0.05) after adjusted for age and sex. The patients aged 50–59 years group had significantly higher *H*. *pylori* infection rate than 30–39 years group (Fig 2). The prevalence of *H*. *pylori* infection among the Protestant was significantly higher than that among Catholic (OR 4.42, P = 0.008). It was also significantly lower among peoples who used tap water as source of drinking water than from Wells/river although after adjusted age and sex. However final model analysis found only ethnics was significantly as independent risk factor for *H*. *pylori* infection (OR = 11.48 [CI 1.12–118.24], OR = 13.32 [CI 1.54–114.96] and OR = 23.47 [2.76–199.51], P <0.05 for Papuan, Batak, Buginese, respectively than Javanese). There were no statistically significant relationship between *H*. *pylori* infection rate and gender, social economic status, type of occupation, marital status, body mass index, type of symptoms, type of latrine, history of drugs, smoking habit and alcohol consumption.

**Table 6:** Association of demographic and sanitation with *H*. *pylori* infection status.

Variable	Total (+*H*. *pylori*%)	Crude OR	95% CI for OR	P
Age				
≤29	8 (20.5%)	1.81	0.54–6.10	0.34
30–39	5 (12.5%)	1.00		
40–49	19 (28.4%)	2.77	0.94–8.14	0.06
50–59	17 (29.8%)	2.98	1.00–8.90	0.05
≥60	10 (15.6%)	1.30	0.41–4.11	0.66
Gender				
Males	32 (25.8%)	1.49	0.84–2.67	0.18
Females	27 (18.9%)	1.00		
Religion				
Muslim	13 (13.8%)	1.36	0.42–4.49	0.61
Catholic	4 (10.5%)	1.00		
Protestant	40 (34.2%)	4.42	1.46–13.32	0.008
Others	2 (11.1%)	1.06	0.18–6.42	
Ethnic				
Javanese	1 (2.4%)	1.00		
Papuan	9 (42.9%)	30.75	3.53–267.68	0.002
Batak	28 (40.0%)	27.33	3.55–210.32	0.001
Buginese	11 (36.7%)	23.74	2.85–197.39	0.003
Dayak	3 (7.5%)	3.32	0.33–33.37	0.31
Tionghoa	7 (13.0%)	6.11	0.72–51.73	0.97
Others	0 (0.0%)	0.0	0.00	0.99
Social economic status				
< Rp.2.500.000 (192.31 USD)	35 (24.3%)	1. 59	0.83–3.03	0.16
Rp.2.500.000–5.000.000	17 (16.8%)	1.00		
> Rp. 5.000.000	7 (31.8%)	2.31	0.82–6.51	0.12
Occupation				
Government job	12 (31.6%)	3.23	0.36–29.28	0.30
Health workers	1 (12.5%)	1.00		
Student	0 (0.0%)	0.00	0.00	0.99
Housewife	12 (16.2%)	1.36	0.15–12.04	0.79
Farmer	15 (46.9%)	6.18	0.68–56.15	0.11
Private job	17 (19.1%)	1.65	0.19–14.35	0.65
Unemployed	2 (16.7%)	1.40	0.11–18.62	0.80
Marital status				
Unmarried	6 (16.7%)	1.00		
Married	53 (22.9%)	1.49	0.59–3.77	0.40
Body Mass Index				
<18.5	5 (17.9%)	1.30	0.13–13.37	0.82
18.5–24.9	44 (25.0%)	2.00	0.23–17.07	0.53
25–29.9	8 (14.5%)	1.02	0.11–9.65	0.99
>30	1 (14.3%)	1.00		
Source of drinking water				
Mineral	26 (17.6%)	3.74	0.48–29.30	0.21
PAM	3 (21.4%)	4.91	0.45–53.27	0.19
Wells/river	29 (35.4%)	9.67	1.23–76.12	0.03
Tap water	1 (4.3%)	1.00		
Latrine				
Toilet	58 (22.0%)	1.00		
Non toilet	1 (33.3%)	1.78	0.16–19.93	0.64
History of drugs				
No	29 (19.0%)	1.64	0.46–5.86	0.45
PPI, H2blockers, antibiotics	27 (30.0%)	3.00	0.83–10.91	0.10
Others	3 (12.5%)	1.00		
Smokers				
Yes	18 (30.0%)	1.64	0.85–3.16	0.14
No	38 (20.8%)	1.00		
Alcohol consumption				
No	41 (20.8%)	1.00		
Yes	15 (32.6%)	1.84	0.91–3.73	0.09

## Discussion

Although culture remains a reference method due to ability to directly detect *H*. *pylori* organisms, it have limited sensitivity. Moreover guideline mentioned there was no single test can be considered as the gold standard for the diagnosis of *H*. *pylori* infection [25]. In the present study, we used four different *H*. *pylori* tests to increase diagnostic accuracy as well as to compare results among tests. We confirmed that the *H*. *pylori* infection prevalence in five largest islands in Indonesia using combination of the diagnostic tests to be 22.1%, contrast with several Southeast Asian countries with high *H*. *pylori* infection prevalence such as Thailand and the Philippines (54.1 to 76.1% and 60%, respectively) [26,27], but almost similar with Malaysia which also low incidence of gastric cancer country. The prevalence of *H*. *pylori* infection in Orang Asli, the aboriginal community, residing in the state of Kelantan, Malaysia has been reported to be 19% [28].

However as a wide country with spans almost 2 million square kilometers between Asia and Australia and consist of 300 distinct native ethnic groups, the prevalence of *H*. *pylori* in Indonesia should be observed by considering ethno-geographic group. Moreover, several ethnics had higher prevalence of *H*. *pylori* infection than Chinese Indonesian, the highest prevalence ethnic was reported in previous study [5]. The highest prevalence of *H*, *pylori* ethnic, Papuans, is various indigenous peoples of Papua island and neighboring islands. They are speakers of the Papuan languages and often distinguished ethnically and linguistically from Austronesians. Most of them quite maintain their traditions, especially who living in central mountainous region/highland zones [29]. The high prevalence of *H*. *pylori* infection in Papuans concordance with previous study which reported the prevalence was 58% in Papua New Guinea, eastern part of Papua island [30]. It will be interesting to know the genotypes of *H*. *pylori* strains of Papuans peoples which may have similarity with New Guinea and Aborigines Australia strains. The eastern sections of Indonesia, especially Papua, were geographically connected to Australia as a single continent (Sahul) about 60,000 years ago.

The lower prevalence of Chinese descent than that of Chinese non-immigrants was reported in previous studies [5,31]. Chow *et al*. reported that seroprevalence of Chinese which born in Malaysia/Singapore (43.1%) were lower than those born in China/Hong Kong (68.2%) [31]. By multivariate analysis they also found that the higher risk for *H*. *pylori* infection in chopsticks users which suggests person-to-person transmission of *H*. *pylori* via the oral-oral route with ethno-specific food practices an important risk factor. Environmental factors might contribute to the lower *H*. *pylori* infection rate in Chinese Indonesians. Beside use Chinese cuisine legacy, Chinese Indonesian also modified some of the dishes with addition of Indonesian local ingredients [32] which might associated with the low prevalence of *H*. *pylori* as same as ‘budu’ or local anchovy sauce, and ‘pegaga’ or centenella asiatica have also been reported to be associated with the low prevalence of *H*. *pylori* in Malaysia [33].

Another interesting result we found was that Buginese, a majority numerous ethnic in the southern part of Sulawesi had also high prevalence of *H*. *pylori* (36.7%), however still lower than that in Philippines [27]. Sulawesi and the Philippines except for Palawan is assumed to be zoogeographical separated with Sundaland (mainland of Asia) which is supported by distributional patterns [34]. Alfred Russell Wallace designated a faunal boundary organisms demarcating the transition between Asian and Melanesian features (Lombok eastwards, Sulawesi, the Moluccas and Philippines-but not Palawan). Most of the languages of the Wallace region belonging to the extensive Austronesian language family but with more distantly related Papuan languages occurring in the Far Eastern provinces, especially in areas where Melanesian features predominate [35]. Contrary with this study, our previous study found the prevalence of *H*. *pylori* infection in North Sulawesi was very low (14.3%) by urine test confirmed with serology [11]. Recently we also confirmed these data with five different diagnostic tests (unpublished data). It is still unclear why there was difference of *H*. *pylori* prevalence within Sulawesi island. We should remark that Sulawesi island consist of various indigenous ethnic groups which have different phenotype. Although we observed hspMaori type in North Sulawesi, a subpopulation of East Asian type, often isolated from native Taiwanese and Maori tribe as well as some subjects in Philippines [36].

The extremely low prevalence of *H*. *pylori* infection in Javanese group also confirmed our previous study in Surabaya, Java island [5]. Javanese had a low *H*. *pylori* prevalence as well as Malay ethnic group in Malaysia which have the similar host genetic factors that reduced susceptibility to *H*. *pylori* infection [37,38]. In the last ice age, the central and western sections of the Indonesian archipelago were connected by dry land to the Asian mainland (Sundaland) including Java, Sumatera and Borneo island. Therefore it is not surprising that Dayak ethnic also had low prevalence of *H*. *pylori* infection. Dayak is the indigenous peoples of Borneo which was categorized on Malayo-Polynesian linguistic subgroup speakers. Europeans created the term `Dayak`to refer to the non-Malay inhabitants of Borneo [39].

However it is still questionable why Batak ethnic in North Sumatera had high prevalence of *H*. *pylori* infection. Genotyping of *H*. *pylori* strains and host factors analysis from Batak ethnic may partly explain the reason of these differences. The transmission routes of *H*. *pylori* are still not entirely understood, but human-to-human spread through oral-oral or faecal-oral routes are considered the most plausible routes for infection [18]. Therefore intra-racial or intra-community spread such as transmission from mother to child might contribute to these racial differences in *H*. *pylori* infection rates.

Our data showed there were difference of several demographic and environmental factors between ethnic groups; age, religion and the source of drinking water were associated with increased risk of infection. Several ethnics showed the age-related prevalence pattern of *H*. *pylori* infection in developing countries that *H*. *pylori* infections occur earlier in life and with high frequency [18]. We also found the prevalence of *H*. *pylori* infection in Protestant was higher than that in Catholic. The religious beliefs and practices might be as important factors for spreading *H*. *pylori* in Indonesia. However it may also due to that in this study, the majority of Protestant were Papuan and Batak ethnics which have the highest prevalence of *H*. *pylori*. The use of well or river water was associated with increased risk of infection. Therefore, consistent with several previous studies [33,40], *H*. *pylori* could survive and contaminate the local water supplies to be the most plausible. However in fact, only ethnics as independent risk factor for *H*. *pylori* infection. Further studies in each group is needed to clarify the most influenced variables of demographic and sanitation which related prevalence patterns of *H*. *pylori* infection in Indonesia, especially in high prevalence area.

Similar with our previous study [5], rapid urease test showed higher positive rate compared with other tests. Compared with histology and culture, RUT is faster, cheaper, and has comparable sensitivity and specificity even in Indonesia. The number of samples in this study were relatively low, certainly become the limitation in this study. In addition, we included only patients with dyspepsia in our study population. In general, the prevalence of *H*. *pylori* infection is higher in dyspeptic patients than in the general population. Currently we are still continuing surveys to add the sample numbers and expand to other islands, including collecting serum. A larger sample size is necessary to elucidate the prevalence of *H*. *pylori* in Indonesia. We just performed survey in 1–2 cities every island. Most of the cities is a capitol of province which may have better sanitary and socio-economic condition than the rural part. Therefore, our results cannot be generalized across Indonesia. A study to investigate genotypes of *H*. *pylori* strains in Indonesia is now in progress. Genotyping information may partly explain the differences of *H*. *pylori* infection among ethnic group in Indonesia.

## Conclusion

Several ethnics group have higher risk for *H*. *pylori* infection than Javanese group, predominantly ethnic which reported have low prevalence of *H*. *pylori* infection in previous study. The age, religion and water source may implicated as a risk factor for *H*. *pylori* infection in Indonesia. Improving the sanitary conditions to decrease the prevalence of *H*. *pylori* in Indonesia is important.

## Supporting Information

S1 Table *H*. *pylori* infections were diagnosed based on the combined results of three methods from four different tests; culture, histology confirmed by immunohistochemistry and rapid urease test. When patients were considered to be *H*. *pylori* positive in case at least one test showed positive, the prevalence of *H*. *pylori* infection was 22.1% (59/267).
(PDF)

## Abbreviations

IHC: immunohistochemistry
RUT: rapid urease test
