# Supplementary material for: Risk Factors and Prevalence of Helicobacter pylori in Five Largest Islands of Indonesia: A Preliminary Study
Source: PLoS One. 2015 Nov 23;10(11):e0140186. doi: 10.1371/journal.pone.0140186 (PMC4658100; doi:10.1371/journal.pone.0140186)
Supplement: S1 Table — When patients were considered to be H. pylori positive in case at least one test showed positive, the prevalence of H. pylori infection was 22.1% (59/267). (PDF) [file pone.0140186.s001.pdf]

Supporting Information Captions

S1 Table. *H. pylori* infections were diagnosed based on the combined results of three methods from four different tests; culture, histology confirmed by immunohistochemistry and rapid urease test. When patients were considered to be *H. pylori* positive in case at least one test showed positive, the prevalence of *H. pylori* infection was 22.1% (59/267).

No	Name of sample		Age	Sex	Rapid urease test	Histology + IHC	Culture	1 of 4
1	JKT	1	57	F	0	0	0	0
2	JKT	2	24	M	0	0	0	0
3	JKT	3	35	M	0	0	0	0
4	JKT	4	36	M	0	0	0	0
5	JKT	5	23	F	0	0	0	0
6	JKT	6	37	F	0	0	0	0
7	JKT	7	41	F	0	0	0	0
8	JKT	8	40	F	0	0	0	0
9	JKT	9	48	M	1	1	1	1
10	JKT	10	34	M	0	0	0	0
11	JKT	11	64	M	0	0	0	0
12	JKT	12	23	M	0	0	0	0
13	JKT	13	38	F	0	0	0	0
14	JKT	14	70	M	0	0	0	0
15	JKT	15	60	F	0	0	0	0
16	JKT	16	55	M	0	0	0	0
17	JKT	20	65	M	0	0	0	0
18	JKT	21	69	F	0	0	0	0
19	JKT	22	29	M	0	0	0	0
20	JKT	23	28	M	0	0	0	0
21	JKT	24	43	M	0	0	0	0
22	JKT	25	42	F	0	0	0	0
23	JKT	26	65	F	0	0	0	0
24	JKT	27	46	F	0	0	0	0
25	JKT	28	61	M	0	0	0	0
26	JKT	29	55	M	0	0	0	0
27	JKT	30	65	F	0	0	0	0
28	JKT	31	73	F	0	0	0	0
29	JKT	32	54	F	0	0	0	0
30	JKT	33	55	F	0	0	0	0
31	JKT	34	62	F	0	0	0	0
32	JAY	1	61	M	1	1	1	1
33	JAY	2	63	M	0	0	0	0
34	JAY	3	25	M	1	1	1	1

35	JAY	4	26	F	1	1	1	1
36	JAY	5	41	M	0	0	0	0
37	JAY	6	41	F	1	1	1	1
38	JAY	7	37	F	0	0	0	0
39	JAY	8	31	F	1	1	1	1
40	JAY	9	41	M	0	0	0	0
41	JAY	10	28	F	0	0	0	0
42	JAY	11	32	M	0	0	0	0
43	JAY	12	34	F	1	1	1	1
44	JAY	13	46	M	0	0	0	0
45	JAY	14	47	F	0	0	0	0
46	JAY	15	59	M	1	1	1	1
47	JAY	16	52	M	0	1	1	1
48	JAY	17	42	M	0	0	0	0
49	JAY	18	23	M	0	0	0	0
50	JAY	19	33	F	0	0	0	0
51	JAY	20	32	F	0	0	0	0
52	JAY	21	48	F	1	1	1	1
53	MDN	1	63	M	0	0	0	0
54	MDN	2	63	F	0	0	0	0
55	MDN	3	69	F	0	0	0	0
56	MDN	4	47	F	0	0	0	0
57	MDN	5	52	M	0	0	0	0
58	MDN	6	29	F	0	0	0	0
59	MDN	7	47	F	0	0	0	0
60	MDN	8	47	F	1	0	0	1
61	MDN	9	74	F	0	0	0	0
62	MDN	10	35	M	0	0	0	0
63	MDN	11	76	M	0	0	0	0
64	MDN	12	39	F	0	0	0	0
65	MDN	13	57	F	0	0	0	0
66	MDN	14	45	F	0	0	0	0
67	MDN	15	80	F	0	0	0	0
68	MDN	16	62	F	0	0	0	0
69	MDN	17	52	M	1	1	1	1
70	MDN	18	60	F	0	0	0	0
71	MDN	19	50	F	0	0	0	0
72	MDN	20	64	F	0	0	0	0
73	MDN	21	54	F	0	0	0	0
74	MDN	22	68	F	0	0	0	0
75	MDN	23	52	F	1	1	1	1
76	MDN	24	40	F	0	0	0	0
77	MDN	25	57	F	0	0	0	0
78	MDN	26	50	M	0	0	0	0
79	MDN	27	27	M	1	1	1	1

80	MDN	28	33	M	0	0	0	0
81	MDN2	1	30	F	0	0	0	0
82	MDN2	2	61	F	0	0	0	0
83	MDN2	3	59	F	1	1	1	1
84	MDN2	4	33	F	0	0	0	0
85	MDN2	5	44	F	0	0	0	0
86	MDN2	6	55	F	0	0	0	0
87	MDN2	8	51	F	0	0	0	0
88	MDN2	9	69	M	0	0	0	0
89	MDN2	10	28	M	1	1	1	1
90	MDN2	11	43	F	1	1	1	1
91	MDN2	12	39	F	0	0	0	0
92	MDN2	13	28	M	0	0	0	0
93	MDN2	15	61	M	1	1	1	1
94	MDN2	16	41	F	1	1	0	1
95	MDN2	17	42	F	0	0	0	0
96	MDN2	18	56	M	0	0	0	0
97	MDN2	19	38	F	1	1	1	1
98	MDN2	20	45	M	1	1	1	1
99	MDN2	21	53	M	0	0	0	0
100	MDN2	22	27	M	1	1	1	1
101	MDN2	23	46	M	1	1	1	1
102	MDN2	24	45	F	1	1	1	1
103	MDN2	25	58	M	1	1	1	1
104	MDN2	26	41	F	1	1	0	1
105	MDN2	27	47	M	0	0	0	0
106	MDN2	28	24	M	0	0	1	1
107	MDN2	29	56	M	1	1	0	1
108	MDN2	30	60	M	1	0	1	1
109	MKS	31	47	M	1	1	1	1
110	MKS	32	59	M	0	0	0	0
111	MKS	33	70	M	0	0	0	0
112	MKS	34	31	M	0	0	0	0
113	MKS	35	62	F	0	0	0	0
114	MKS	36	68	F	0	0	0	0
115	MKS	37	36	F	0	0	0	0
116	MKS	38	34	F	1	0	0	1
117	MKS	39	24	M	1	0	0	1
118	MKS	40	74	F	1	0	0	1
119	MKS	41	30	M	0	0	0	0
120	MKS	42	36	M	0	0	0	0
121	MKS	43	49	M	0	0	0	0
122	MKS	44	43	F	0	0	0	0
123	MKS	45	29	M	1	1	1	1
124	MKS	46	46	F	0	0	0	0

125	MKS	47	46	M	1	1	1	1
126	MKS	48	41	F	0	0	0	0
127	MKS	49	22	F	0	0	0	0
128	MKS	50	72	F	0	0	0	0
129	MKS	51	56	M	0	1	0	1
130	MKS	52	58	F	0	0	1	1
131	MKS	53	33	F	0	0	0	0
132	MKS	54	65	M	0	0	0	0
133	MKS	55	76	M	1	1	1	1
134	MKS	56	67	F	1	1	1	1
135	MKS	57	67	M	1	0	0	1
136	MKS	58	67	M	0	0	0	0
137	MKS	59	41	M	0	0	0	0
138	MKS	60	50	F	0	0	0	0
139	PTN	61	22	F	0	0	0	0
140	PTN	62	27	F	0	0	0	0
141	PTN	63	50	F	1	1	1	1
142	PTN	64	64	F	0	0	0	0
143	PTN	65	54	M	0	0	0	0
144	PTN	66	42	M	0	0	0	0
145	PTN	67	64	F	0	0	0	0
146	PTN	68	41	M	0	0	0	0
147	PTN	69	39	M	0	0	0	0
148	PTN	70	32	M	0	0	0	0
149	PTN	71	42	F	0	0	0	0
150	PTN	72	29	F	0	0	0	0
151	PTN	73	26	M	0	0	0	0
152	PTN	74	44	M	0	0	0	0
153	PTN	75	49	M	1	1	1	1
154	PTN	76	49	M	0	0	0	0
155	PTN	77	60	F	0	0	0	0
156	PTN	78	59	F	0	0	0	0
157	PTN	79	29	F	0	0	0	0
158	PTN	80	48	M	0	0	0	0
159	PTN	81	41	M	0	0	0	0
160	PTN	82	29	M	0	0	0	0
161	PTN	83	77	M	0	0	0	0
162	PTN	84	32	M	0	0	0	0
163	PTN	85	52	F	0	0	0	0
164	PTN	86	25	M	0	0	0	0
165	PTN	87	18	M	0	0	0	0
166	PTN	88	61	M	0	0	0	0
167	PTN	89	70	F	0	0	0	0
168	PTN	90	51	F	0	0	0	0
169	PTN	91	54	M	0	0	0	0

170	PTN	92	41	M	1	0	0	1
171	PTN	93	59	F	0	0	0	0
172	PTN	94	41	M	0	0	0	0
173	PTN	95	20	M	0	0	0	0
174	PTN	96	50	F	0	0	0	0
175	PTN	97	50	F	0	0	0	0
176	PTN	98	55	M	0	0	0	0
177	PTN	99	23	F	0	0	0	0
178	PTN	100	18	M	0	0	0	0
179	PTN2	1	77	M	0	0	0	0
180	PTN2	2	64	F	0	0	0	0
181	PTN2	3	48	M	0	0	0	0
182	PTN2	4	25	F	0	0	0	0
183	PTN2	5	31	M	0	0	0	0
184	PTN2	6	46	M	0	0	0	0
185	PTN2	7	58	F	0	0	0	0
186	PTN2	8	61	F	0	0	0	0
187	PTN2	9	64	F	0	0	0	0
188	PTN2	10	49	F	0	0	0	0
189	PTN2	11	59	F	0	0	0	0
190	PTN2	12	63	M	0	0	0	0
191	PTN2	13	55	M	1	0	0	1
192	PTN2	14	54	F	0	0	0	0
193	PTN2	15	32	M	0	0	0	0
194	PTN2	16	56	F	0	0	0	0
195	PTN2	17	42	M	0	0	0	0
196	PTN2	18	58	F	0	0	0	0
197	PTN2	19	64	M	0	0	0	0
198	PTN2	20	35	F	1	1	1	1
199	PTN2	21	74	M	0	0	0	0
200	PTN2	22	33	M	0	0	0	0
201	PTN2	23	41	M	0	1	0	1
202	PTN2	24	70	M	0	0	0	0
203	MDN3	1	36	F	0	0	0	0
204	MDN3	2	34	M	0	0	0	0
205	MDN3	3	47	M	0	0	0	0
206	MDN3	4	52	M	0	1	0	1
207	MDN3	5	56	F	1	1	0	1
208	MDN3	6	43	M	0	0	0	0
209	MDN3	7	49	M	1	0	0	1
210	MDN3	8	43	F	1	0	0	1
211	MDN3	9	43	F	1	0	0	1
212	MDN3	10	58	M	1	1	0	1
213	MDN3	11	65	F	0	0	0	0
214	MDN3	12	46	F	1	0	0	1

215	MDN3	13	48	F	0	0	0	0
216	MDN3	14	57	F	1	0	0	1
217	MDN3	15	50	F	1	0	0	1
218	SBY	105	41	F	0	0	0	0
219	SBY	106	67	F	1	1	1	1
220	SBY	107	51	M	1	0	0	1
221	SBY	108	34	F	0	0	0	0
222	SBY	109	64	M	0	0	0	0
223	SBY	110	26	M	0	0	0	0
224	SBY	111	44	F	0	0	0	0
225	SBY	112	64	M	0	0	0	0
226	SBY	113	27	F	0	0	0	0
227	SBY	114	23	F	0	0	0	0
228	SBY	115	52	F	0	0	0	0
229	SBY	116	46	M	0	0	0	0
230	SBY	117	49	F	0	0	0	0
231	SBY	118	50	F	0	0	0	0
232	SBY	119	46	M	0	0	0	0
233	SBY	120	61	F	0	0	0	0
234	SBY	121	53	M	0	0	0	0
235	SBY	122	48	F	0	0	0	0
236	SBY	123	60	F	0	0	0	0
237	SBY	124	60	F	0	0	0	0
238	SBY	125	37	F	0	0	0	0
239	SBY	126	63	F	0	0	0	0
240	SBY	127	22	F	0	0	0	0
241	SBY	128	39	F	0	0	0	0
242	SBY	129	23	M	0	0	0	0
243	SBY	130	71	F	0	0	0	0
244	SBY	131	57	F	0	0	0	0
245	SBY	132	62	M	0	0	0	0
246	SBY	133	54	M	0	0	0	0
247	SBY	134	44	F	0	0	0	0
248	SBY	135	55	M	0	0	0	0
249	SBY	136	49	M	0	0	0	0
250	SBY	137	61	F	1	0	1	1
251	SBY	138	55	F	0	0	0	0
252	SBY	139	22	F	0	0	0	0
253	SBY	140	53	F	0	0	0	0
254	SBY	141	25	M	0	0	0	0
255	SBY	142	70	F	0	0	0	0
256	SBY	143	47	F	0	0	0	0
257	SBY	144	17	F	0	0	0	0
258	SBY	145	39	M	0	0	0	0
259	SBY	146	18	F	0	0	0	0

260	SBY	147	49	F	0	0	0	0
261	SBY	148	49	F	0	0	0	0
262	SBY	149	56	M	0	0	0	0
263	SBY	150	39	F	0	0	0	0
264	SBY	151	77	M	1	1	1	1
265	SBY	152	69	M	0	0	0	0
266	SBY	153	32	M	0	0	0	0
267	SBY	154	39	F	0	0	0	0
